# Integrated PANoptosis Profiling Identifies Immunosuppressive Subtypes and a Prognostic Signature With Functional Validation of MLKL in Glioblastoma

**DOI:** 10.1002/acn3.70481

**Published:** 2026-07-08

**Authors:** Langfei Tian, Minghui Zhao, Yuanbo Hu, Kaiyue Wang, Haiguang Liu, Zetong Bai, Kebin Zheng

**Affiliations:** ^1^ Department of Neurosurgery The Affiliated Hospital of Hebei University Baoding Hebei Province China; ^2^ School of Clinical Medicine Hebei University Baoding Hebei Province China

**Keywords:** glioblastoma, MLKL, PANoptosis, prognosis

## Abstract

**Objective:**

The prognosis of glioblastoma (GBM) remains highly unfavorable, largely due to high tumor heterogeneity and an immunosuppressive microenvironment. However, the functional role of PANoptosis in this context is poorly understood.

**Methods:**

Patients were stratified via K‐means clustering. A risk score model was constructed using prognosis‐associated genes identified by Cox regression and validated in independent cohorts. Immune infiltration was analyzed using CIBERSORT and ESTIMATE. Single‐cell RNA sequencing (scRNA‐seq) profiled the tumor microenvironment. Functional assays were performed following MLKL knockdown.

**Result:**

Two molecular subtypes based on PANoptosis‐related genes were identified, with distinct survival and immune features. A five‐gene (MLKL, YWHAG, GZMB, ELANE, CASP4) risk score served as an independent prognostic factor. The high‐risk group exhibited an inflamed yet dysfunctional tumor immune microenvironment, marked by higher PD‐L1 expression, T cell dysfunction, and Merck18 score. scRNA‐seq confirmed elevated activity of PANoptosis in GBM. Finally, MLKL knockdown was shown to suppress malignant phenotypes and induce apoptosis.

**Conclusion:**

Our findings link PANoptosis to GBM heterogeneity, providing a prognostic model and nominating MLKL as a key functional mediator, which may inform patient stratification and the development of targeted therapies.

## 1 Introduction

Glioblastoma (GBM) is a highly aggressive tumor of the central nervous system with a poor prognosis [1, 2]. Current treatments, including surgery, radiotherapy, and chemotherapy, offer only limited control, contributing to poor patient outcomes [3]. This is due to the inherent biology of GBM: significant tumor heterogeneity, a highly immunosuppressive tumor microenvironment (TME), and therapy resistance driven by GBM stem cells (GSCs) [4, 5, 6, 7, 8]. Therefore, deciphering novel mechanisms of its progression and identifying new therapeutic targets are urgently needed.

Programmed cell death (PCD) is crucial for the homeostasis of tissues and elimination of abnormal cells [9, 10]. Beyond classical apoptosis, novel forms of PCDs like pyroptosis, necroptosis, and ferroptosis have emerged, forming a complex network of cell death. PANoptosis, a newly defined inflammatory PCD integrating features of pyroptosis, apoptosis, and necroptosis [11], has drawn significant attention for its immunoregulatory potential. PANoptosis‐related genes (PANRGs) are aberrantly expressed in various cancers, and their expression patterns correlate with immune cell infiltration, inflammatory status in the TME, and patient prognosis [12]. In peripheral tumors like melanoma and colorectal cancer, targeting PANoptosis via drug combinations or specific molecules shows potential to overcome chemoresistance and boost anti‐tumor immunity [13, 14, 15, 16, 17]. These findings indicate that targeting PANoptosis could be a multi‐dimensional strategy to remodel the tumor immune microenvironment (TIME).

In GBM, which has an immune‐privileged environment, the role of PANoptosis, its mechanisms in shaping the immune microenvironment, and the utility of its key molecules as prognostic markers or therapeutic targets remain unclear. Recent studies suggest that targeting PANoptosis is a novel strategy to improve GBM therapy, with potential in three areas: first, multi‐omics‐based PANoptosis scores or gene models can predict patient prognosis and distinguish TIME [18, 19]; second, specific compounds like cinobufagin can induce PANoptosis, trigger immunogenic cell death, promote anti‐tumor immunity, and convert “cold” tumors to “hot” ones, thereby enhancing the efficacy of immunotherapies [20]; third, cytokines such as tumor necrosis factor‐α and interferon (IFN)‐γ exert anti‐tumor effects by inducing PANoptosis [21, 22]. These advances highlight the clinical need to systematically elucidate the regulatory network of PANoptosis in GBM for developing new combination therapies.

Based on this background, this study explores the regulatory role and clinical significance of PANoptosis in GBM. Using The Cancer Genome Atlas (TCGA), the Chinese Glioma Genome Atlas (CGGA), and the Gene Expression Omnibus (GEO) databases, we performed bioinformatics analysis on reported PANRGs to develop and validate a risk score model. This analysis identified mixed lineage kinase domain‐like protein (MLKL), a key necroptosis executor, as a core candidate. In vitro experiments confirmed that MLKL regulates the malignancy of GBM cells and the release of inflammatory cytokines. From the novel perspective of PANoptosis, this study not only establishes MLKL as a promising independent prognostic biomarker but also reveals its role in the progression of GBM by regulating the crosstalk between cell death and immune signaling. This provides a new theoretical basis and a potential target for developing precise combination therapeutic strategies against GBM.

## 2 Methods and Materials

### 2.1 Data Acquisition and Preprocessing

Expression profiles of genes, clinical data, and survival information for GBM were obtained from TCGA (https://portal.gdc.cancer.gov/) database. After excluding duplicates and selecting primary cases with complete survival information, 169 TCGA‐GBM samples were retained; count data were converted to transcripts per million (TPM). The GSE43378 dataset (*n* = 50), providing microarray expression and clinical data, was sourced from the Gene Expression Omnibus (GEO). The CGGA mRNAseq‐693 dataset (FPKM‐normalized) was downloaded from the Chinese Glioma Genome Atlas (CGGA, http://www.cgga.org.cn/), and 176 GBM samples with complete survival data were selected. FPKM values were similarly transformed to TPM. As each dataset was used independently for training or validation, batch correction was not applied. For external validation (CGGA and GSE43378), expression of the five model genes was *Z*‐score normalized using the mean and standard deviation of the TCGA training set to ensure consistent input. Differential expression between GBM and normal brain tissues was analyzed using the Gene Expression Profiling Interactive Analysis (GEPIA, http://gepia.cancer‐pku.cn/) database. Additionally, basal MLKL expression in GBM cell lines was validated using the Cancer Cell Line Encyclopedia (CCLE, https://sites.broadinstitute.org/ccle) database.

### 2.2 Unsupervised Clustering

Sixty‐six PANRGs were selected from the literature [23]. Using the “ConsensusClusterPlus” R package (k‐means algorithm), unsupervised consensus clustering was performed on GBM RNA‐seq data. The analysis included 1000 iterations with 80% resampling to ensure stability. The optimal number of clusters (k = 2) was determined by evaluating the consensus matrix and cumulative distribution function (CDF) curve via the “NbClust” package. Samples were stratified into two groups (Cluster 1 vs. Cluster 2). Overall survival (OS) between clusters was compared using Kaplan–Meier analysis with the log‐rank test (*p* < 0.05).

### 2.3 Gene Ontology (GO) and Kyoto Encyclopedia of Genes and Genomes (KEGG) Enrichment Analysis

Following stratification into Cluster 1 and Cluster 2, differentially expressed genes (DEGs) were identified using the “limma” R package. The threshold criteria were set at |log_2_FoldChange| ≥ 1 and adjusted *p*‐value (FDR) < 0.05. Functional annotation of significant DEGs was conducted using the “clusterProfiler”. This encompassed a three‐tiered classification of GO terms: molecular function (MF), biological process (BP), and cellular component (CC), alongside KEGG analysis. The uniform cutoff of significance was set at FDR < 0.05.

### 2.4 Construction and Validation of the Risk Score Model

Univariate Cox regression on 66 PANRGs in TCGA‐GBM identified prognosis‐associated genes, which were further refined via least absolute shrinkage and selection operator (LASSO) Cox with 10‐fold cross‐validation to mitigate overfitting and multicollinearity, ultimately establishing a robust five‐gene risk score model. Details are as follows:

$$\begin{array}{c} \text{Risk Score}=\left(0.1163\times \text{MLKL expression}\right)\\ +\left(0.5753\times \text{YWHAG expression}\right)+\left(0.5348\times \text{GZMB expression}\right)\\ +\left(0.2832\times \text{ELANE expression}\right)+\left(0.1867\times \text{CASP4 expression}\right) \end{array}$$

The median risk score served as the cutoff to stratify subject cohorts into two (high‐risk score [HRS] and low‐risk score [LRS]) groups. Kaplan–Meier analysis with the log‐rank test was used to compare OS between groups. The discriminative power of the model in predicting survival outcomes at 1, 3, and 5 years was evaluated using time‐dependent receiver operating characteristic (ROC) analysis implemented with the “timeROC” R package. For external validation, the generalizability and stability of the model were assessed using the independent datasets GSE43378 and CGGA.

### 2.5 Correlation Between the Risk Score Model and Clinical Factors

To evaluate the association between the risk score and clinical characteristics, both univariate and multivariate Cox proportional hazards regression analyses were performed. Subsequently, variables for the nomogram were selected based on the results of the multivariate Cox regression. Variables that were statistically significant independent predictors (*p* < 0.05) in the multivariate model were included to construct a parsimonious and clinically applicable nomogram using the “rms” R package. The predictive accuracy of this nomogram was validated by plotting calibration curves to assess the consistency between the model‐predicted survival outcomes and the actual observed results.

### 2.6 Analysis of Tumor Immune Microenvironment (TIME)

Immune infiltration heterogeneity across subgroups was characterized using the CIBERSORT algorithm (http://cibersortx.stanford.edu/). Spearman correlation analysis evaluated the associations between risk score and the abundance of specific immune cell subsets. ESTIMATE‐derived scores (Stromal, Immune, and ESTIMATE) were compared between groups. The tumor immune dysfunction and exclusion (TIDE) framework was used to assess the immune evasion potential.

### 2.7 Gene Set Enrichment Analysis (GSEA)

Differential expression analysis between high‐ and low‐risk groups was conducted with DESeq2, after filtering low‐count genes. Genes were ranked by log2FoldChange. Using this ranked list, Gene Set Enrichment Analysis (GSEA) was performed for GO and KEGG using the “gseGO” and “gseKEGG” functions from the “clusterProfiler” package. Key parameters were: 1000 permutations, minimum gene set size = 10, maximum size = 1000, and *p*‐value cutoff = 0.05. Significantly enriched results (*p* < 0.05) were visualized using the “enrichplot” and “GseaVis” packages.

### 2.8 Drug Sensitivity Analysis

Drug sensitivity was analyzed using the “oncoPredict” R package, which predicts the half‐maximal inhibitory concentration (IC_50_) for each sample using a GDSC2‐trained model. The Wilcoxon rank‐sum test compared IC_50_ values between high‐ and low‐risk groups to identify agents with significant sensitivity differences (*p* < 0.05). Pearson correlation analyzed the relationship between key risk gene expression and the IC_50_ of filtered drugs. Results were visualized via bubble plots, with size and color indicating correlation strength and significance. All analyses were performed in R using “oncoPredict”, “psych”, and “ggplot2”.

### 2.9 Immunophenoscore (IPS) Analysis

To evaluate tumor immunogenicity, the immunophenoscore (IPS) data from TCGA were analyzed. The four IPS subsets, representing different combinatorial expression states of CTLA4 and PD‐1, were compared between high‐ and low‐risk groups using the non‐parametric Wilcoxon rank‐sum test. Results were visualized with the “ggplot2” and “ggpubr” R packages.

### 2.10 Single‐Cell RNA Sequencing (scRNA‐Seq) Analysis

This study analyzed scRNA‐seq data from the GSE162631 dataset, which contains 3 GBM tumor samples and 3 normal brain tissue samples, to compare GBM with normal tissues using the “Seurat” R package. Stringent quality control (QC) was applied: cells were retained if they expressed between 200 and 6000 genes and had mitochondrial gene content below 10% (Figure 6A). Data normalization was performed using the LogNormalize method, and the top 2000 highly variable genes were identified for downstream analysis. To mitigate batch effects, data integration was performed using the Harmony algorithm after Principal Component Analysis (PCA).

Unsupervised cell clustering was conducted using a shared nearest neighbor graph and the Louvain algorithm (resolution = 0.5) (Figure 6B). Cell clusters were annotated based on established marker genes (Figure 6C,D): microglia (CX3CR1, P2RY12), macrophages (APOC1), proliferating macrophages (TOP2A, MKI67), T cells (CD3D, CD3E), endothelial cells (CLDN5, VWF), mural cells (RGS5, PDGFRB), dendritic cells (HLA‐DQA1), and neutrophils (IL1R2, CXCR2). Finally, the expression patterns of core genes from a risk score were analyzed across the identified cell types and between groups. All visualizations were generated using the R packages “Seurat”, “ggplot2”, and “ggpubr”.

### 2.11 Cell Culture and Transfection

U343 and U118MG cell lines (Procell, Wuhan, China) were incubated at 37°C in complete medium (DMEM, 10% FBS) with 5% CO_2_. Small interfering RNAs (siRNAs) targeting MLKL (siMLKL#1, #2, #3), a negative control (si‐NC), and a positive control (si‐PC) were synthesized by Genepharma (Shanghai, China). Cells were transfected with siRNAs and GP‐TransFectmate (Cat. #G04008; Genepharma, Shanghai, China) per the manufacturer's instructions. Before proceeding to further experiments, cells were cultured for 24–48 h. Identical transfections were performed on cells seeded in separate plates, with the quantity of all materials proportionally adjusted. GAPDH served as the positive control gene. The siRNA sequences are provided in Table 1.

**TABLE 1:** SiRNAs sequences of MLKL, negative control, and positive control.

iMLKL#1	Sense	AGAGAUGAAAUACUGCAAGAA (dT) (dT)
	Antisense	UUCUUGCAGUAUUUCAUCUCU (dT) (dT)
siMLKL#2	Sense	CGCUGUUACUUCAGGUUGA (dT) (dT)
	Antisense	UCAACCUGAAGUAACAGCG (dT) (dT)
siMLKL#3	Sense	GAAGCUUCACUGAGACGAUUA (dT) (dT)
	Antisense	UAAUCGUCUCAGUGAAGCUUC (dT) (dT)
si‐NC	Sense	UUCUCCGAACGUGUCACGU (dT) (dT)
	Antisense	ACGUGACACGUUCGGAGAA (dT) (dT)
si‐PC	Sense	GUCAUCAGUCUCCACGGAGA (dT) (dT)
	Antisense	UCCGUUGACUCCGACCTACA (dT) (dT)

### 2.12 Real‐Time Quantitative PCR (RT‐qPCR)

Following the manufacturer's instructions, total RNA was isolated from U343 and U118MG cells using Trizol reagent (Invitrogen, Carlsbad, CA, USA). One microgram of RNA was reverse transcribed into cDNA using the Geneseed II First Strand cDNA Synthesis Kit (Geneseed, GS0201‐2) under the following conditions: 25°C for 15 min, 98°C for 5 min, and a final hold at 4°C. For RT‐qPCR, the Geneseed qPCR SYBR Green Master Mix (GS0201‐3, Geneseed) kit was applied on a real‐time PCR system (7500, ABI). The specific cycling conditions were as follows: 5 min at 95°C, then 40 repeated cycles of 10 s at 95°C and 34 s at 60°C. A final dissociation step at 95°C for 1 min was included. GAPDH was used as the reference gene. mRNA expression was analyzed via the 2^−ΔΔCt^ method. Primer information is detailed in Table 2.

**TABLE 2:** Real‐time PCR primer sequences used in this study.

Gene	Primer	Sequence (5′‐3′)	PCR
IL‐1α	Forward	TTGGCGTTTGAGTCAGCAAA	162 bp
	Reverse	CATGGAGTGGGCCATAGCTT	
HMGB1	Forward	CAGAACAGAAATACATCTCAGGGC	165 bp
	Reverse	TCGTGCACCGAAAGTTTCAA	
GAPDH	Forward	GAAAGCCTGCCGGTGACTAA	113 bp
	Reverse	GCATCACCCGGAGGAGAAAT	
MLKL	Forward	GCCACCGAGACGATTAGA	182 bp
	Reverse	CCAGTCTGACATCTTCAC	

### 2.13 Western Blot and Enzyme‐Linked Immunosorbent Assay (ELISA)

Proteins were isolated from cells using RIPA buffer. Their concentrations were determined by the BCA assay. Equal amounts of protein were subjected to SDS‐PAGE and transferred onto PVDF membranes. The membranes were incubated at 4°C overnight with primary antibodies against MLKL (1:2000; ET1601‐25, HuaBio, China), p‐MLKL (1:2000; 82,090–2‐RR, Proteintech, China), interleukin (IL)‐1α (1:1000; ab9722, Abcam, UK), and β‐actin (1:2000; 20,536–1‐AP, Proteintech, China). Then, they were incubated with secondary antibodies (1:10000; SA00001‐2, Proteintech, China) for 50 min. Protein bands were visualized using enhanced chemiluminescence (ECL) and scanned by an Aplegen Omega LumC chemiluminescent gel imaging system.

For the detection of secreted HMGB1, cell culture supernatants were collected 48 h after siRNA transfection. The concentration of HMGB1 was measured using a human HMGB1 ELISA kit (CSB‐E08223h; Cusabio Biotech, Wuhan, China) according to the manufacturer's instructions. Absorbance was read at 450 nm.

### 2.14 Cell Counting Kit‐8 (CCK‐8) Assay

Following siRNA treatment, U343 and U118MG cells were seeded in 96‐well plates at 1 × 10^4^ cells/well. CCK‐8 reagent (Cat. #40203ES60, YEASEN) and 100 μL of DMEM medium were added at 10 μL/well. The plates were then incubated for 1 h. Absorbance values were measured at 450 nm by a plate reader (Thermo, Multiscan MK3).

### 2.15 Wound Healing Assay

A total of 5 × 10^5^ cells were seeded into 6‐well plates and cultured overnight to achieve sufficient confluence. A sterile tip was used to create a scratch across the cell layer. Detached cells were washed away with phosphate‐buffered saline (PBS). Fresh DMEM medium was added for continued incubation. Images were captured under a microscope (×40 magnification) at 0, 24, and 48 h.

### 2.16 Plate Colony Formation Assay

Viable cells were plated at 1000 cells/well into 6‐well plates and cultured for 7 or 14 days at 37°C with 5% CO_2_. The medium was removed. Cells were rinsed twice with PBS, fixed for 15 min with 4% paraformaldehyde, rinsed with PBS once more, stained for 10 min with crystal violet, subjected to an additional PBS rinse, and then allowed to air‐dry under ambient conditions. Colonies were imaged and counted to calculate the colony formation rate.

### 2.17 Flow Cytometry for Apoptosis Detection

Single‐cell suspensions were prepared by trypsinization (without EDTA) followed by washing with ice‐cold PBS. Apoptosis was evaluated utilizing Annexin V‐FITC/PI double staining (MultiSciences Biotech). Data were acquired using a flow cytometer (Novocyte D2060R). Finally, quantitative analysis was performed using FlowJo software (FlowJo, Ashland, USA).

### 2.18 Statistical Methods

Statistical computations and visualization were implemented in R (v4.5.2) and GraphPad Prism (v10). The experiments described in this article were repeated three times independently. Data were analyzed using two‐tailed Student's *t*‐test or one‐way ANOVA for group comparisons, Pearson correlation analysis for associations, and Kaplan–Meier analysis with the log‐rank test and Cox proportional hazards regression models for survival outcomes. Significance cutoffs were defined as follows: ns (not significant); * (*p* < 0.05); ** (*p* < 0.01); *** (*p* < 0.001); **** (*p* < 0.0001).

## 3 Results

### 3.1 Molecular Subtype Identification Based on PANRGs Expression Profiles and Their Immune Microenvironment Characteristics

**FIGURE 1:**
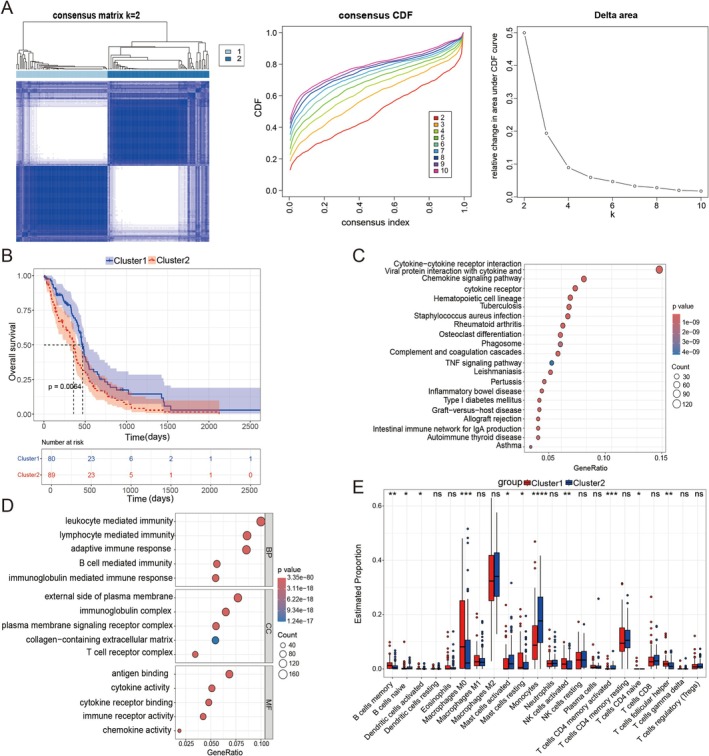
Identification of PANoptosis‐related molecular subtypes and their prognostic and immune microenvironment characteristics. (A) Patients were stratified into two subtypes using consensus clustering (*k* = 2); (B) Kaplan–Meier analysis with the log‐rank test of OS in the two subtypes; (C, D) GO and KEGG enrichment analyses of DEGs between the subtypes; (E) Immune cell infiltration comparison analyzed by CIBERSORT.

Based on the expression profiles of 66 PANRGs, patients were stratified into two subtypes by consensus clustering (Figure 1A). Kaplan–Meier analysis with the log‐rank test revealed that Cluster 2 had significantly shorter OS than Cluster 1, indicating a subtype with poor prognosis (Figure 1B). CIBERSORT analysis revealed that the two subtypes differed significantly in immune cell infiltration (e.g., macrophages, monocytes) (Figure 1E), linking the PANRGs‐driven subtypes to distinct immune microenvironment features. Functional enrichment analysis of DEGs between subtypes showed significant enrichment in immune response (Figure 1C,D), inflammatory response, and related pathways, corroborating the immune infiltration findings.

### 3.2 Construction and Validation of the Risk Score Model

**FIGURE 2:**
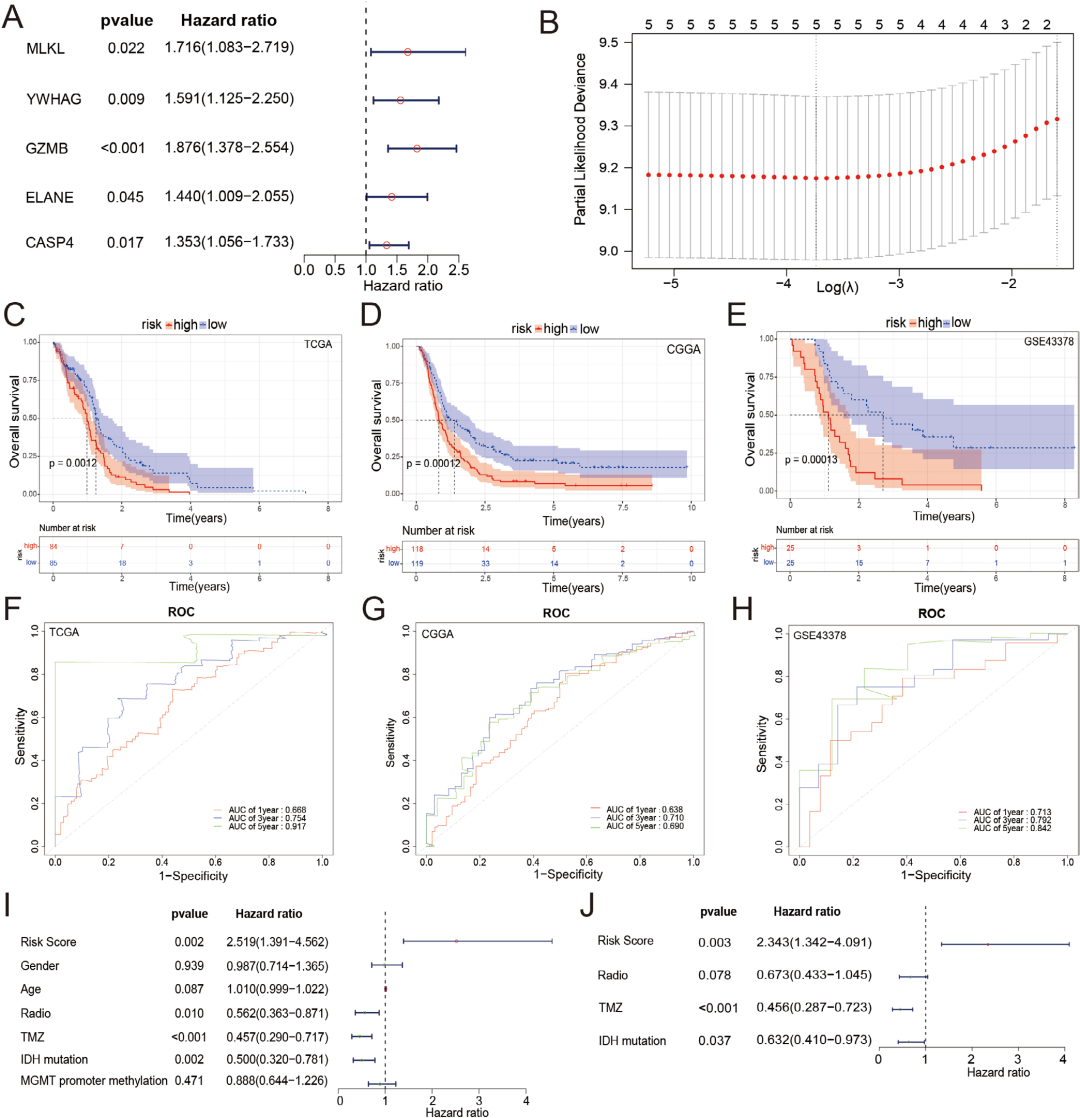
Prognostic prediction for GBM patients using a multigene risk score model based on five genes. (A, B) Identification of prognosis‐associated genes via univariate Cox regression (A) and LASSO regression (B); (C–E) Kaplan–Meier analysis of OS with the log‐rank test comparing high‐ and low‐risk groups in the TCGA (C), CGGA (D), and GSE43378 (E) cohorts; (F–H) ROC curves for predicting 1‐, 3‐, and 5‐year survival using the risk score model in the TCGA training set (F), CGGA validation cohort (G), and GSE43378 validation cohort (H); (I‐J) Univariate (I) and multivariate (J) Cox regression analyses to evaluate the independent prognostic value of the risk score.

Univariate Cox regression of 66 PANRGs identified five prognosis‐associated genes (MLKL, YWHAG, GZMB, ELANE, CASP4; Figure 2A), confirmed by LASSO‐Cox regression using the optimal lambda (Figure 2B). A risk score was derived from their expression levels and coefficients. In the TCGA training set, ROC curve analysis demonstrated good predictive performance for 1‐, 3‐, and 5‐year survival (Figure 2F). Patients were dichotomized into high‐risk and low‐risk groups by the median risk score. Kaplan–Meier analysis with the log‐rank test revealed significantly shorter OS in the HRS group (Figure 2C). The prognostic value of the model was validated in two independent cohorts (CGGA and GSE43378; Figure 2G,H), where the HRS group consistently exhibited poorer survival (Figure 2D,E). Both univariate and multivariate Cox analyses confirmed the risk score as an independent prognostic predictor after adjusting for age, sex, and IDH mutation status (Figure 2I,J).

### 3.3 Construction of a Clinical Nomogram and Characterization of Underlying Genomic Features

**FIGURE 3:**
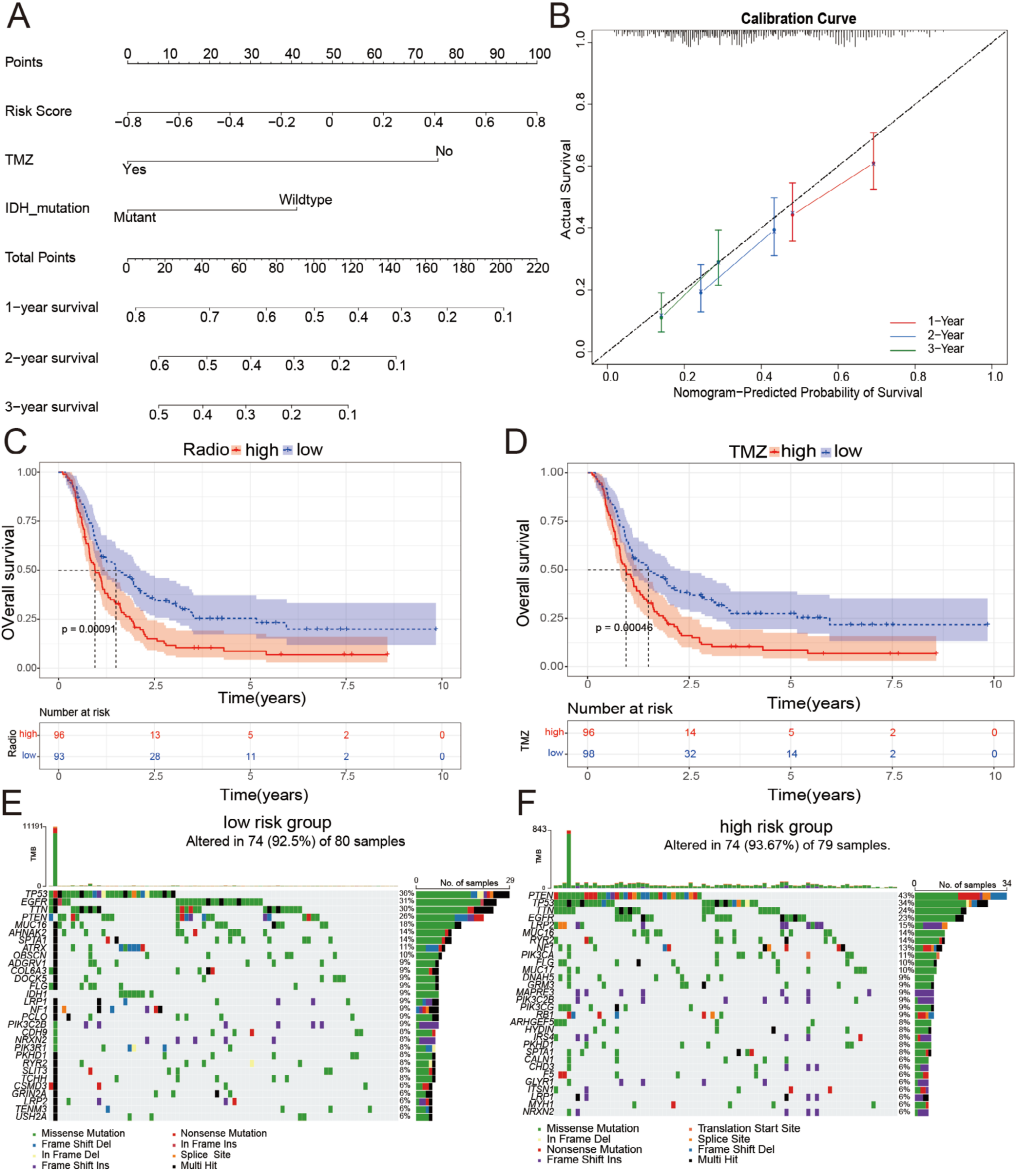
Predictive efficacy and genomic characterization of the nomogram model based on risk score and clinical factors in GBM patients. (A) Nomogram for survival prediction integrating risk score, TMZ treatment, and IDH mutation status; (B) Calibration curves of the nomogram model for predicting 1‐, 2‐, and 3‐year survival rates; (C) Comparison of OS between HRS and LRS groups among patients receiving radiotherapy; (D) Comparison of OS between HRS and LRS groups among patients receiving TMZ treatment; (E) High‐frequency mutation gene spectrum in GBM patients of the LRS group; (F) High‐frequency mutation gene spectrum in GBM patients of the HRS group.

Multivariate Cox analyses confirmed the risk score, temozolomide (TMZ) treatment, and IDH mutation status as independent prognostic factors (Figure 2J). A nomogram integrating these factors predicted 1‐, 2‐, and 3‐year survival (Figure 3A), with calibration curves showing good agreement (Figure 3B). Subgroup analysis showed that among patients receiving radiotherapy or TMZ, the HRS group had significantly shorter OS than the LRS group (Figure 3C,D), indicating that the risk score maintained its prognostic value in these clinically relevant treatment subgroups. Tumor mutational burden analysis revealed comparable overall mutation frequencies (HRS: 93.67%; LRS: 92.50%) but distinct spectra: PTEN (43%), TP53 (34%), and TTN (24%) dominated the HRS group, whereas TP53 (36%), EGFR (31%), and TTN (30%) characterized the LRS group (Figure 3E,F).

### 3.4 Correlation Analysis Between Risk Score and TIME

**FIGURE 4:**
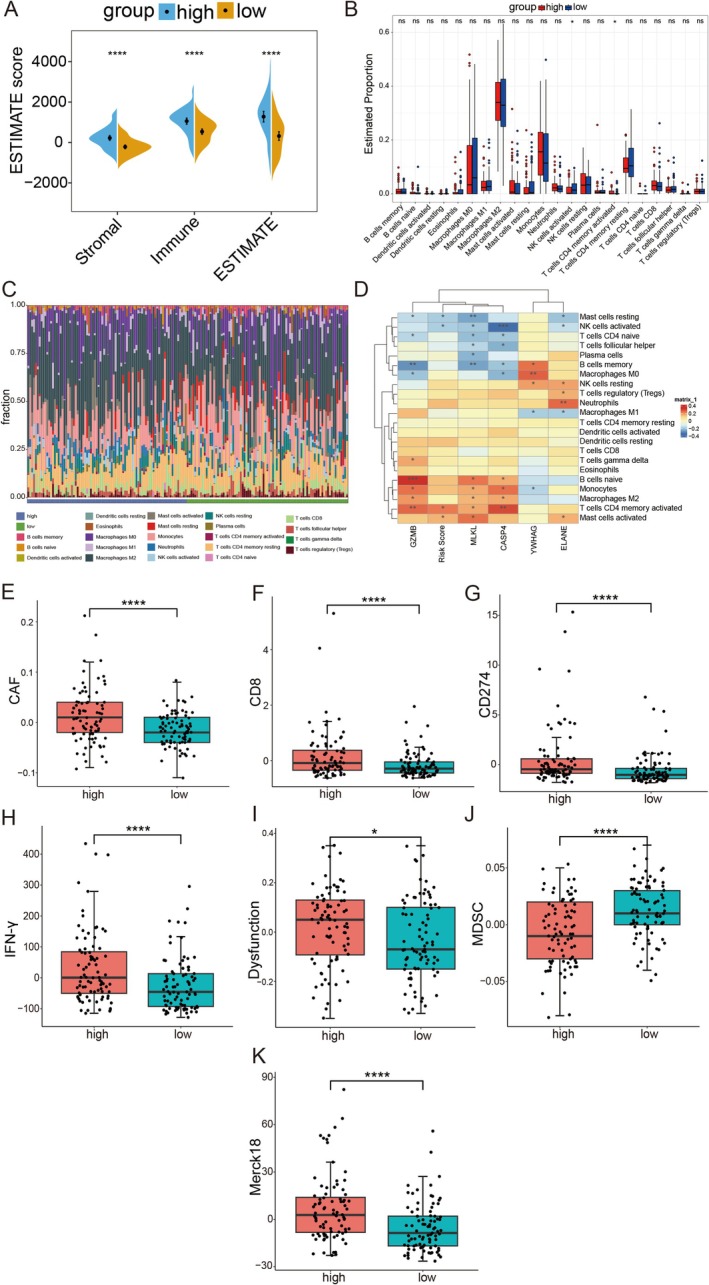
Correlation analysis of the risk score with TIME characteristics and immunotherapy response. (A) Significantly elevated tumor microenvironment scores in the HRS group; (B, C) CIBERSORT analysis of immune cell differential infiltration between HRS and LRS groups; (D) Correlation of the risk score with specific immune cell subsets and the co‐expression network of key genes; (E‐I) The HRS group exhibits an immune‐activated yet T‐cell dysfunctional microenvironment; (J) High infiltration of MDSCs characterizes the LRS group; (K) The HRS group shows a higher predicted immunotherapy response score (Merck18).

The ESTIMATE, immune, stromal, and combined ESTIMATE scores were significantly higher in the HRS group than the LRS group (Figure 4A). CIBERSORT analysis revealed significant differences between groups in the proportions of activated memory CD4^+^ T cells and activated natural killer (NK) cells (Figure 4B,C). The risk score positively correlated with activated memory CD4^+^ T cells and activated mast cells, but negatively correlated with resting mast cells and activated NK cells. Key genes GZMB, MLKL, and CASP4 showed positive correlations, forming a co‐expression module (Figure 4D). TIDE analysis indicated the HRS group had higher infiltration of cancer‐associated fibroblasts (CAFs) (Figure 4E), increased infiltration of CD8^+^ T cells, elevated expression of CD274 (PD‐L1), and elevated IFN‐γ signaling (Figure 4F–H), but also a higher T cell dysfunction score (Figure 4I). In contrast, the LRS group showed higher myeloid‐derived suppressor cells (MDSCs) infiltration (Figure 4J). The Merck18 score for predicting immunotherapy response was significantly higher in the HRS group (Figure 4K).

### 3.5 Functional Enrichment and Therapeutic Response Prediction Analysis of Risk Stratification

**FIGURE 5:**
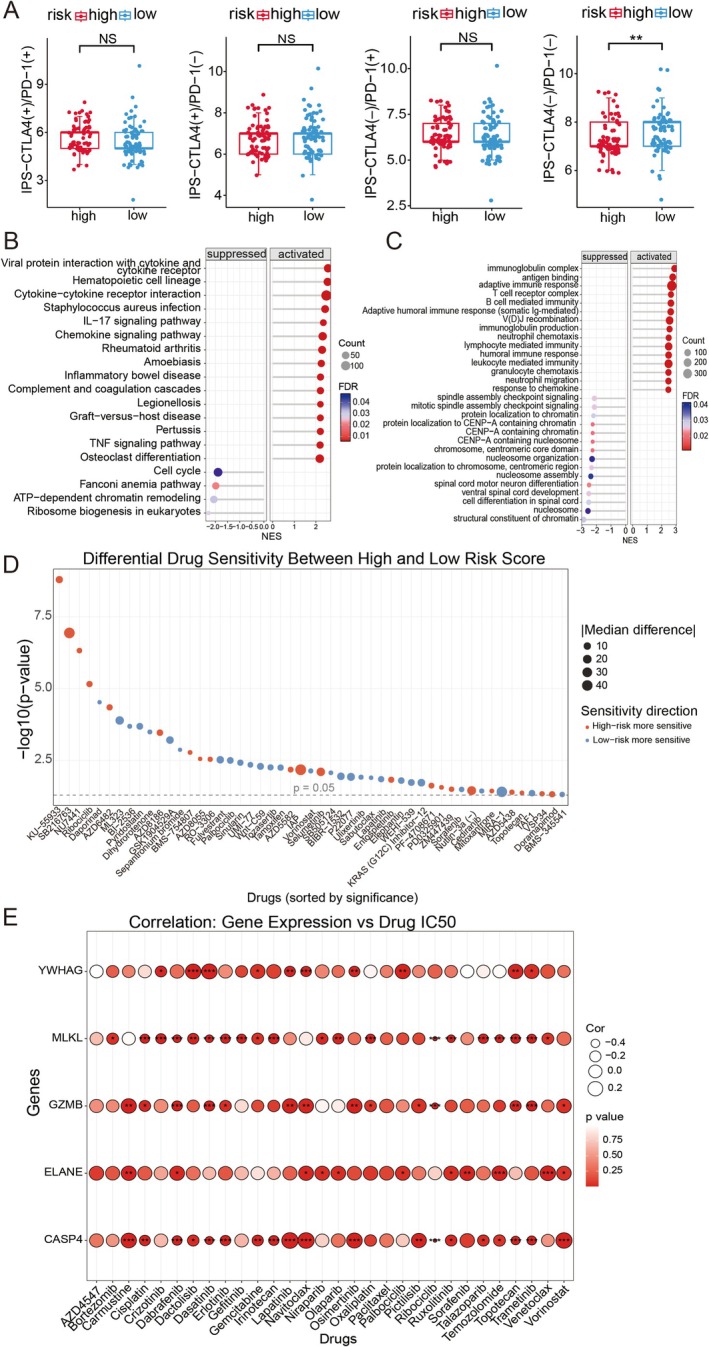
The high‐risk signature is associated with an inflamed TME and distinct therapeutic responses. (A) Comparison of IPS scores between risk groups under different immune checkpoint expression states; (B) Enrichment of the HRS group in immune‐inflammatory KEGG pathways; (C) Enrichment of the HRS group in immune response‐related GO biological processes; (D) Differential sensitivity to specific compounds between HRS and LRS groups; (E) Correlation analysis between key gene expression of the risk signature and sensitivity to commonly used anticancer drugs.

IPS analysis revealed that the score was significantly lower in the HRS group only within the CTLA4(−)/PD‐1(−) subgroup (Figure 5A), indicating that patients in this “cold” immune microenvironment may derive limited benefit from anti–PD‐1/CTLA4 monotherapy and thus warrant combination strategies. GSEA of this subgroup showed significant enrichment of immune‐inflammatory pathways (e.g., “Cytokine–cytokine receptor interaction”, “IL–17 signaling”) and downregulation of “Cell cycle” in the HRS group (Figure 5B). GO analysis similarly enriched “adaptive immune response” (Figure 5C), reflecting dysregulated immune activation consistent with an “inflamed but dysfunctional” microenvironment. To assess clinical potential, drug sensitivity analysis identified significantly different responses: KU‐5933 and SB2‐16763 were more effective in the HRS group, while daporinad and pyridostatin were more effective in the LRS group (Figure 5D). Correlation analysis of the five risk genes with IC_50_ values of 30 anticancer drugs revealed that the expression of YWHAG and MLKL was negatively correlated with IC_50_ values for multiple drugs (e.g., crizotinib, TMZ), whereas the expression of GZMB, ELANE, and CASP4 was positively correlated with IC_50_ values for crizotinib and sorafenib (Figure 5E).

### 3.6 Single‐Cell Transcriptomic Analysis of Risk Model Gene Expression and TME Cellular Landscape in GBM

**FIGURE 6:**
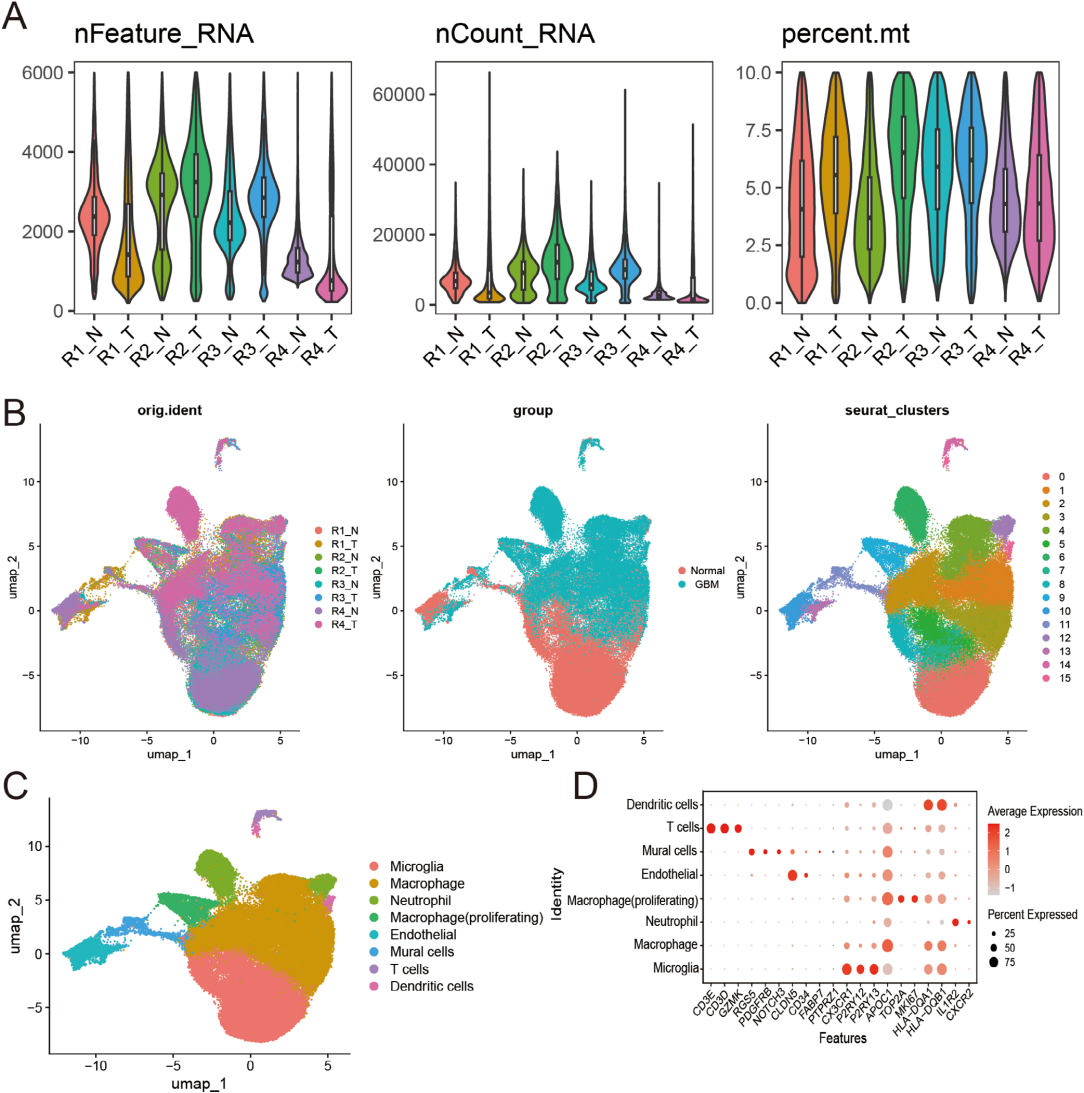
Identification of cell‐type‐specific gene expression patterns through integrated scRNA‐seq data analysis. (A) Data quality control and filtering; (B) Unsupervised clustering reveals cellular heterogeneity; (C) Annotation defines distinct cell types; (D) Cell‐type‐specific differential gene expression.

**FIGURE 7:**
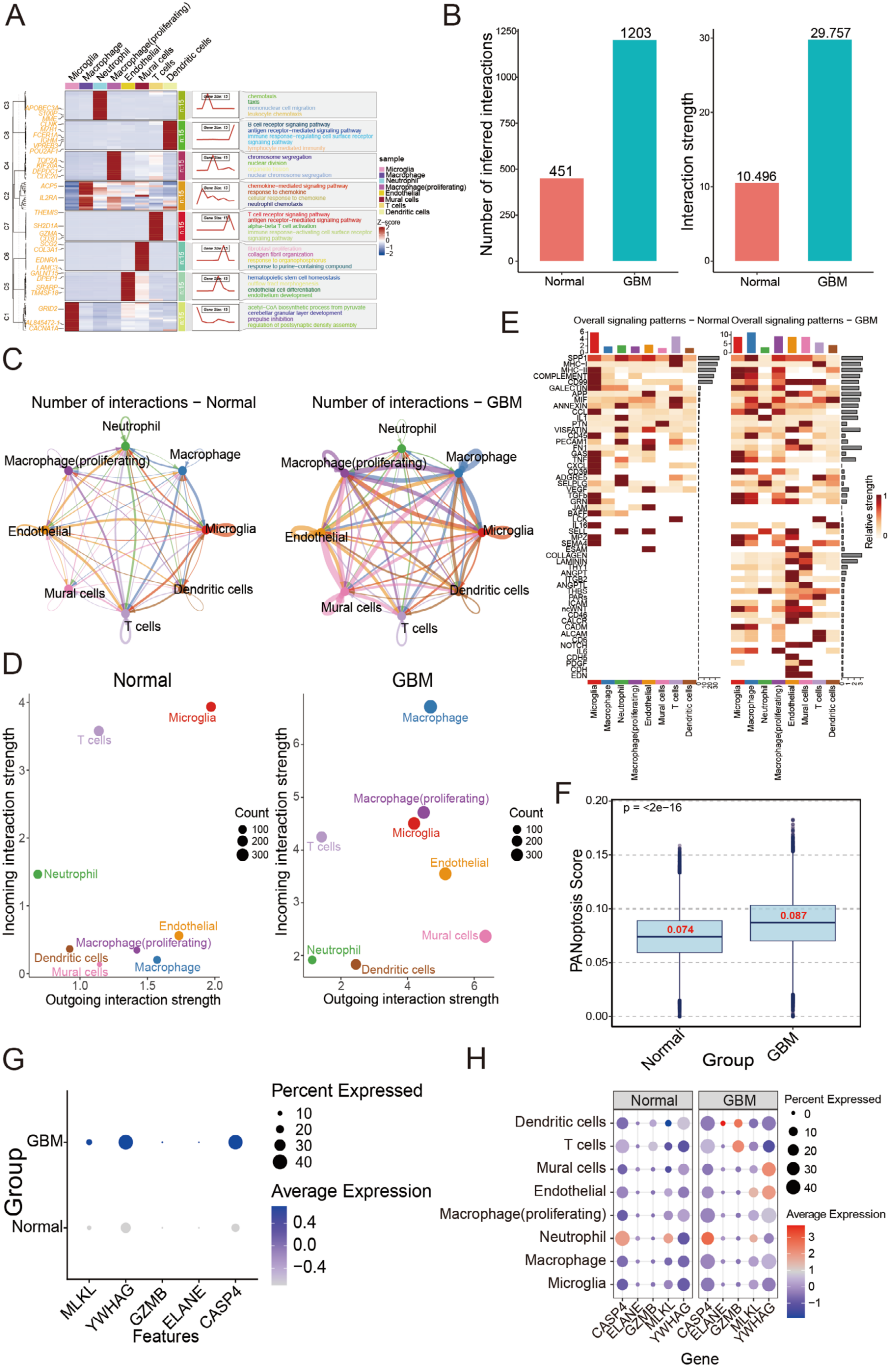
Single‐cell transcriptomics deciphers immune microenvironment reprogramming and cell–cell communication network in GBM. (A) GO functional enrichment pathways of different cell types in GBM versus normal groups; (B) Comparison of inferred number and strength of cell–cell interactions between GBM and normal groups; (C) Cell communication network diagrams of GBM and normal groups; (D) Activity of different cell types as signal senders and receivers in GBM versus control tissues; (E) Differences in global signaling pathway activity between GBM and normal groups; (F) Comparison of PANoptosis scores between GBM and normal groups; (G) Average expression levels of risk signature genes in GBM versus normal groups; (H) Expression proportion of risk signature genes across distinct cell types in GBM and normal groups.

To verify the distribution of core risk genes and cellular interactions in the TME of GBM, scRNA‐seq data from GBM and normal tissues were analyzed. After quality control and clustering, ten major cell types were identified (Figure 6C,D). GO enrichment revealed significant enrichment of inflammatory and immune pathways in microglia, macrophages, and T cells in GBM (Figure 7A). Cell–cell communication analysis showed substantially more and stronger interactions in GBM, forming myeloid‐centered signaling networks (Figure 7B–E).

Mapping the PANoptosis gene set to the scRNA‐seq data demonstrated significantly elevated activity in GBM versus normal cells (*p* < 0.05, Figure 7F). The five risk genes (MLKL, YWHAG, GZMB, ELANE, CASP4) exhibited higher average expression and broader distribution across macrophages, T cells, and endothelial cells in GBM (Figure 7G,H).

### 3.7 Clinical Significance and Expression Validation of the Key Gene MLKL

**FIGURE 8:**
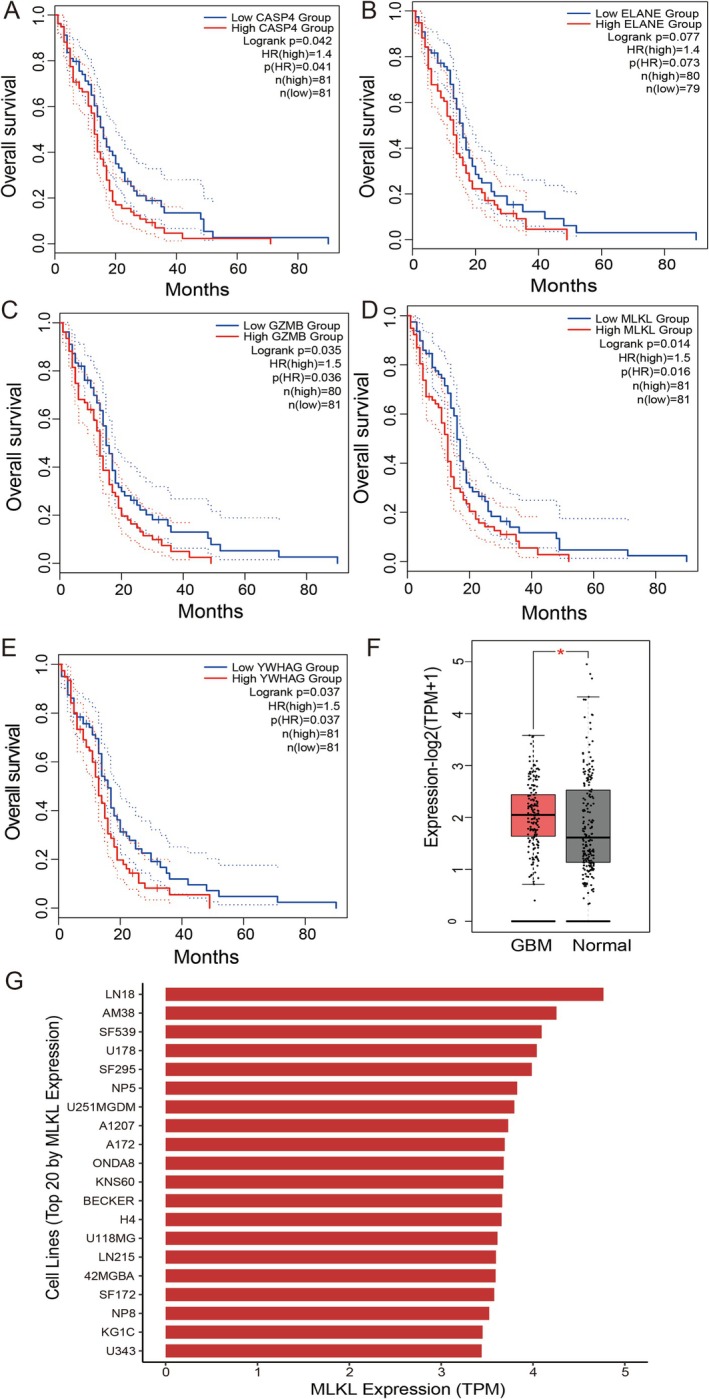
Screening and validation of the core gene MLKL in GBM. (A–E) Kaplan–Meier analysis with the log‐rank test to evaluate the prognostic impact of CASP4, ELANE, GZMB, MLKL, and YWHAG on GBM patients; (F) Differential expression of MLKL between GBM and normal tissues; (G) Expression levels of MLKL across a panel of patient‐derived GBM cell lines.

Kaplan–Meier analysis with the log‐rank test of the five risk genes revealed that high expression of MLKL, CASP4, GZMB, and YWHAG was significantly associated with poor prognosis (Figure 8A–E). Given the central role of MLKL in the PANoptosis pathway, we focused on it for subsequent investigation. Analysis using the GEPIA database confirmed that the expression of MLKL was significantly higher in GBM tissues compared to normal brain tissues (Figure 8F). Data from the CCLE database indicated that this gene was expressed in various GBM cell lines (Figure 8G). Based on the expression profiles of laboratory‐available cell lines, U343 and U118MG cell lines were selected, which exhibited high expression of MLKL for subsequent functional experiments.

### 3.8 Impact of MLKL Knockdown on the Malignant Phenotype of GBM Cells

**FIGURE 9:**
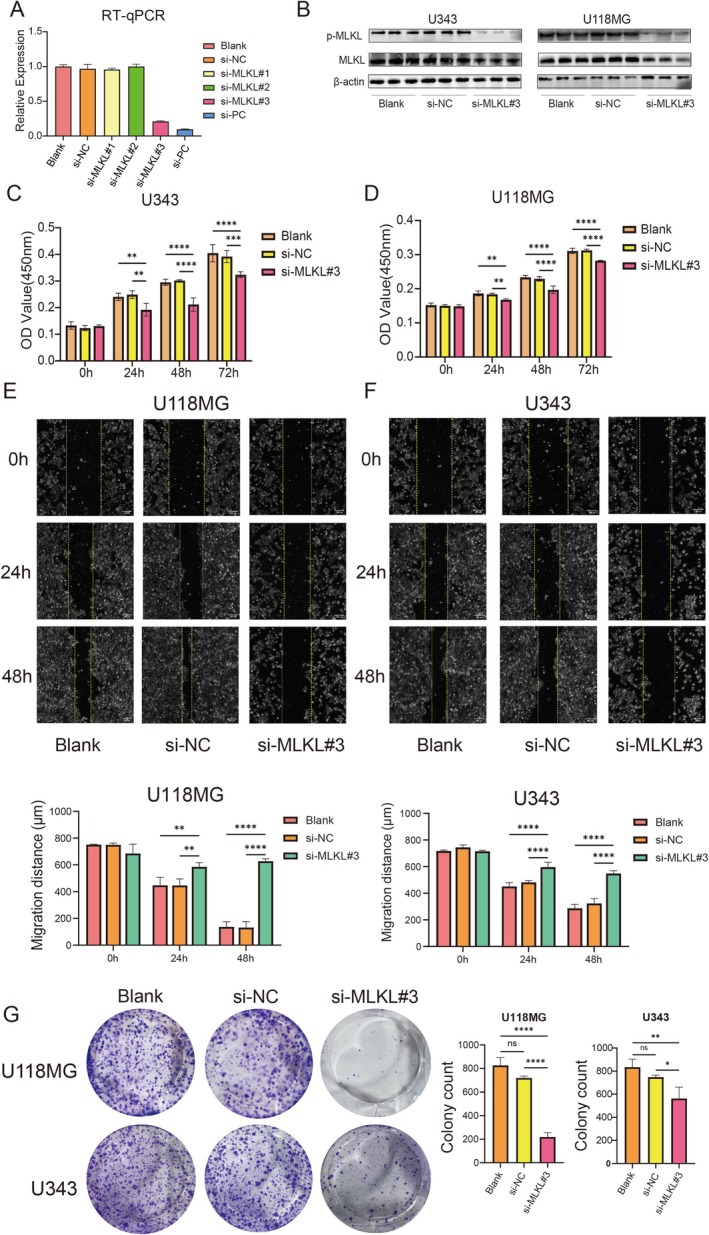
Targeting MLKL attenuates key oncogenic behaviors in GBM. (A, B) Successful silencing of MLKL expression was achieved using specific siRNAs, as verified by RT‐qPCR and Western blot; (C‐D) Cell viability assessed by CCK‐8 assay was significantly reduced in U343 (C) and U118MG (D) cells upon MLKL knockdown; (E‐F) Wound healing assays demonstrated markedly impaired migratory ability in U118MG (E) and U343 (F) cells following MLKL silencing; (G) MLKL knockdown reduces clonogenic potential.

To investigate the functional role of MLKL, three specific siRNAs were designed and transfected into U343 cells. qRT‐PCR validation showed that si‐MLKL#3 had the highest knockdown efficiency (Figure 9A) and was therefore selected for subsequent experiments. Western blot analysis further confirmed that si‐MLKL#3 effectively reduced the protein levels of both MLKL and its phosphorylated form (p‐MLKL) in U343 and U118MG cells (Figure 9B). Functional assays demonstrated that knockdown of MLKL significantly inhibited cell viability (Figure 9C,D), delayed wound healing (Figure 9E,F), and reduced colony formation (Figure 9G).

### 3.9 MLKL Knockdown Affects Inflammatory Factor Expression and Cell Apoptosis

**FIGURE 10:**
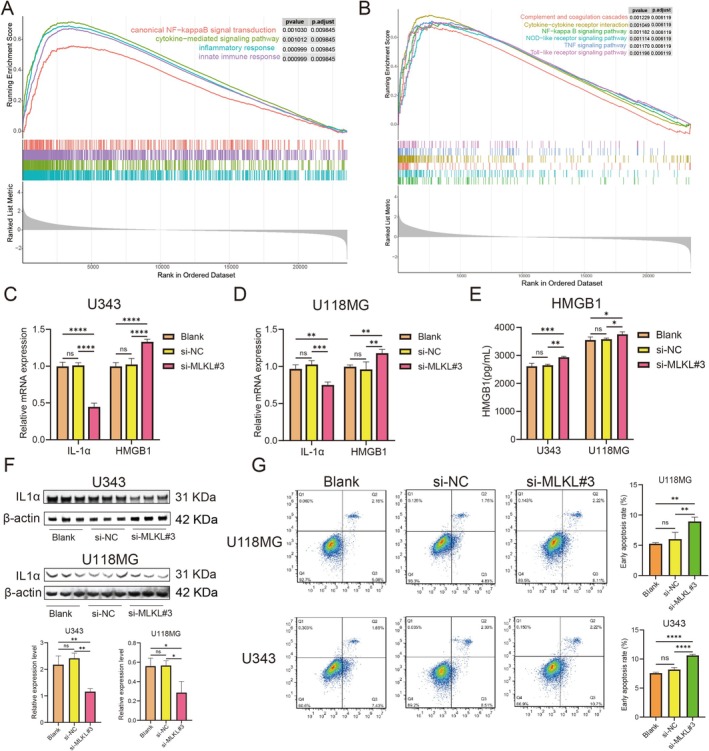
MLKL regulates inflammatory factor expression and influences cell apoptosis. (A, B) GSEA analysis indicating that high MLKL expression impacts inflammatory signaling pathways; (C, D) RT‐qPCR showing altered IL‐1α and HMGB1 expression in U343 and U118MG cells after MLKL knockdown; (E) ELISA quantification of secreted HMGB1 in cell culture supernatants after MLKL knockdown; (F) Western blot analysis of intracellular IL‐1α protein levels in U343 and U118MG cells after MLKL knockdown; (G) Flow cytometry demonstrating the effect of MLKL knockdown on early apoptosis in U118MG and U343 cells.

GSEA based on the expression of MLKL revealed significant enrichment of inflammatory pathways in the group with a high expression of MLKL (Figure 10A,B), consistent with its established role in inflammatory signaling. In U343 and U118MG cells transfected with si‐MLKL#3, RT‐qPCR showed that MLKL knockdown significantly suppressed IL‐1α mRNA but upregulated HMGB1 (Figure 10C,D). ELISA confirmed increased secretion of HMGB1 in the supernatant (Figure 10E). Western blot further demonstrated decreased intracellular IL‐1α protein levels (Figure 10F), corroborating the mRNA results. These findings indicated that MLKL differentially regulated inflammatory factors at both transcriptional and protein levels. Moreover, flow cytometry revealed that MLKL knockdown significantly increased early apoptosis in both GBM cell lines (Figure 10G).

## 4 Discussion

By integrating multi‐omics data, this study constructed a PANoptosis‐based molecular classification and prognostic risk model for GBM, highlighting its central role in pathogenesis and its link to TIME heterogeneity and therapeutic response.

Based on the expression profiles of PANRGs, GBM patients were classified into two subtypes with distinct survival outcomes. This aligns with the trend of high‐resolution GBM subtyping using multi‐dimensional features, such as subtypes defined by immune interaction networks [24], a single‐cell multi‐omics “tumor ecosystem” framework [25], and high‐risk subtypes identified via integrated multi‐modal radiomics [26]. These studies highlight GBM heterogeneity from multiple angles. The PANRG‐based classification revealed a distinct immune infiltration pattern, with DEGs enriched in immune and inflammatory pathways. This suggests that PANRGs are not just cell death regulators but also shape the GBM immune microenvironment, supporting the view that the immunosuppressive microenvironment is co‐shaped by tumor cells and myeloid cells [4, 27]. Notably, single‐cell communication analysis revealed enhanced microglia/macrophage signaling hubs, implicating PANRGs in the reprogramming of the TIME and offering a “cell death‐immune crosstalk” perspective on GBM heterogeneity.

The risk score model based on five PANRGs (MLKL, YWHAG, GZMB, ELANE, CASP4) showed strong prognostic predictive ability across multiple independent cohorts and was validated as an independent prognostic indicator, independent of traditional factors such as IDH mutation status. This aligns with trends in cancer research, where similar PANRG models in other cancers also effectively predict prognosis and immune features [28, 29]. These studies affirm that concise multi‐gene signatures from core biological processes, converted into quantitative risk scores, are a clinically powerful strategy. Notably, unlike previous glioma studies [18, 20], the current model specifically targets GBM and integrates genes related to pyroptosis/necroptosis (MLKL, CASP4), granzyme‐mediated apoptosis (GZMB), and neutrophil inflammation (ELANE). This suggests that the high‐risk phenotype may be linked to a more active “PANoptosis” phenotype—an emerging concept of inflammatory PCD integrating multiple death modalities.

TIME analysis unveiled a paradox in the HRS group: high immune/stromal scores, CD8^+^ T cell infiltration, PD‐L1 expression, and IFN‐γ signaling coexisting with elevated T cell dysfunction scores. This “immune‐enriched yet dysfunctional” phenotype offers novel insights into GBM immunotherapy resistance. The discrepancy between a high Merck18 score (strong immunogenicity) and a low IPS score in the CTLA4(−)/PD‐1(−) subgroup (predicting poor monotherapy response) reflects an “inflamed yet immunosuppressed” state, wherein immune recognition is counteracted by the dysfunction of active T cells. Therefore, patients with HRS are unlikely to benefit from single‐agent ICIs but may be ideal candidates for combination regimens that simultaneously reverse immunosuppression and augment effector T‐cell function. This phenomenon parallels CAF‐tumor‐associated macrophage (TAM)‐mediated immunosuppression observed in intrahepatic cholangiocarcinoma [30].

Single‐cell transcriptomic analysis validated the risk model and provided mechanistic insights. PANoptosis activity was confirmed to be elevated in GBM, with the five hub genes widely distributed across myeloid, T cell, and endothelial compartments, indicating that the risk score reflects a multicellular signaling state rather than a purely tumor‐intrinsic property. Cell communication analysis further revealed more intricate intercellular networks in GBM, with myeloid cells functioning as central signaling hubs. This multicellular distribution and enhanced myeloid communication provide a cellular basis for the concurrent immune infiltration and T cell dysfunction observed in the high‐risk group, suggesting that PANoptosis‐related signaling within these subsets may drive the inflamed yet immunosuppressive microenvironment. These findings align with the known roles of TAMs and MDSCs in GBM immunosuppression and imply that PANRGs may contribute to immune dysregulation by modulating myeloid‐T cell crosstalk [31, 32, 33].

The knockdown of MLKL, the necroptosis executor, inhibited the proliferation, migration, and clonogenicity of GBM cells and induced apoptosis. Importantly, it differentially regulated key inflammatory factors like IL‐1α and HMGB1. This is significant because IL‐1α is a pro‐inflammatory cytokine, and HMGB1 is a damage‐associated molecular pattern that amplifies immune responses. MLKL's regulation of these factors may directly shape the TME's inflammatory state and anti‐tumor immunity. This may explain the “high‐inflammation yet immunosuppressive” phenotype in high‐risk patients: MLKL signaling might drive a chronic, pro‐tumorigenic inflammation rather than effective anti‐tumor immunity. Therefore, targeting MLKL could both inhibit tumor growth and potentially reshape the TIME to favor immunotherapy.

This study has several limitations. First, the risk model derived from retrospective bioinformatics requires prospective validation in larger cohorts. Second, although MLKL knockdown suppressed malignant phenotypes in vitro, the lack of in vivo tumorigenesis experiments precludes definitive confirmation of its functional role in GBM. Third, single‐cell sequencing provided preliminary insights, yet the mechanisms by which PANRGs regulate immune subsets need further elucidation using refined approaches such as cell‐specific knockout. Finally, the signaling networks through which MLKL modulates inflammatory factors and the TIME warrant deeper characterization.

Future directions include: (i) integrating the PANRGs risk score with existing GBM molecular subtypes (e.g., classical, mesenchymal, proneural) or immune classifications to build a refined prognostic and predictive system; (ii) validating whether targeting MLKL in preclinical models can enhance the efficacy of radiotherapy, chemotherapy, or immune checkpoint inhibitors (e.g., anti‐PD‐1), especially for identified high‐risk subgroups; and (iii) investigating the roles of other PANRGs (e.g., GZMB, CASP4) in GBM and their potential synergistic or compensatory relationships with MLKL to inform combination targeting strategies.

## 5 Conclusion

This study elucidates the critical role of PANRGs in GBM prognosis, immune microenvironment heterogeneity, and tumor cell malignancy. The constructed five‐gene risk score model serves as a reliable and independent prognostic indicator. Mechanistic investigations identified MLKL as a key functional gene, driving GBM progression by regulating cell proliferation, cell death, and inflammatory cytokine secretion. These findings deepen the understanding of the interplay between cell death and immunity in GBM, providing a valuable tool for prognostic assessment and a theoretical foundation for novel combination therapies, such as targeting PANoptosis to enhance immunotherapy responses.

## Author Contributions

**Langfei Tian:** writing – review and editing, writing – original draft, visualization, methodology, investigation, formal analysis, data curation, conceptualization. **Minghui Zhao:** visualization, methodology, investigation, formal analysis, data curation. **Yuanbo Hu:** software, investigation, formal analysis, data curation. **Kaiyue Wang:** methodology, investigation, formal analysis, data curation. **Haiguang Liu:** visualization, methodology, investigation, formal analysis, data curation. **Zetong Bai:** investigation, formal analysis, data curation. **Kebin Zheng:** resources, supervision, methodology, funding acquisition, conceptualization. All authors commented on previous versions and approved the final manuscript.

## Funding

This research was supported by the Natural Science Foundation of Hebei Province (No. H2023201032), the Scientific Research Project of the Affiliated Hospital of Hebei University (No. 2023ZA02), and the Joint Project of Hebei Provincial Department of Finance and Hebei Provincial Health Commission (No. ZF2026434).

## Ethics Statement

The authors have nothing to report.

## Consent

The authors have nothing to report.

## Conflicts of Interest

The authors declare no conflicts of interest.

## Data Availability

The data that support the findings of this research are available from the corresponding author, K. Zheng, upon reasonable request.
